# Application of Nanoliposome Alprostadil in the Perioperative Period of Percutaneous Coronary Intervention to Reduce In-Stent Restenosis: A Systematic Review and Meta-Analysis

**DOI:** 10.1155/2023/4100197

**Published:** 2023-05-18

**Authors:** Decai Zhu, Dawei Wang, Zhen Zhao, Qingqing Liu, Rongyuan Yang, Qing Liu

**Affiliations:** ^1^The Second Clinical School of Medicine, Guangzhou University of Chinese Medicine, Guangdong Provincial Hospital of Chinese Medicine, Guangdong Provincial Hospital of Chinese Medicine-Zhuhai Hospital, Guangzhou 510120, Guangdong, China; ^2^The First Affiliated Hospital of Guangzhou University of Traditional Chinese Medicine, Guangzhou 510405, Guangdong, China

## Abstract

**Background:**

In-stent restenosis (ISR) is a common complication after percutaneous coronary intervention (PCI) surgery for patients with coronary atherosclerotic heart disease (CHD). Reports indicate alprostadil may reduce ISR, and this study aimed at reviewing and summarizing the effect of nanoliposome alprostadil on ISR by meta-analysis.

**Methods:**

Articles were searched from databases, and meta-analysis was performed in Review Manager software. Funnel plots were performed to evaluate the publication bias, and sensitivity analysis was performed to determine the robustness of the overall treatment effects.

**Results:**

Initially, 113 articles were identified, and 5 studies of 463 subjects were included for analysis eventually. The primary endpoint, i.e., the occurrence of ISR after PCI, occurred in 11.91% of the alprostadil treatment group (28 from 235 patients) vs. 21.49% of the conventional treatment group (49 from 228 patients) and showed a statistical significance in our pooled data (*χ*^2^ = 7.654, *P*=0.006), while there was no statistically significant difference in all of the separate studies. We observed no statistical methodological heterogeneity among the studies (*P*=0.64, *I*^2^ ≈ 0%). The pooled odds ratio (OR) of the occurrence of ISR was 49% in a fixed-effect model, and the 95% confidence boundary (95% CI) was 29% to 81%. The funnel plot did not show serious publication bias, and sensitivity analysis showed well robustness of the overall treatment effect. *Discussion.* In conclusion, the early application of nanoliposome alprostadil after PCI could effectively reduce the occurrence of ISR, and the overall effect of alprostadil treatment in reducing ISR after PCI was relatively stable.

## 1. Introduction

Coronary atherosclerotic heart disease (CHD) is usually caused by coronary atherosclerosis, which leads to narrowing or occlusion of blood vessels, resulting in myocardial ischemia, hypoxia, and necrosis [1]. Percutaneous coronary intervention (PCI) is widely used in the treatment of CHD, but PCI usually injures the vascular endothelium, induces or aggravates vascular inflammatory response, and leads to postoperative in-stent restenosis (ISR) [2]. During the perioperative period of PCI, patients are routinely given antiplatelet, lipid-lowering, antihypertensive, hypoglycemic, and other symptomatic treatments, which is called the regular plan of “the secondary prevention and treatment of coronary heart disease.” Besides, most of the patients after PCI would be given the hydration treatment especially the one with impaired renal function, in order to prevent the occurrence of contrast agent (used in PCI)-induced renal dysfunction. Thus, these treatments were normally used in the clinical studies in the control group. Even though the occurrence rate of ISR after PCI is still high, it needs to be reduced in order to improve the clinical efficacy and prognosis of CHD patients after PCI.

Nanoliposome, or submicron bilayer lipid vesicle, is a new technology for the encapsulation and delivery of bioactive agents. Prostaglandin E1 (PGE1, also named alprostadil), especially its nanoliposome dosage form, was reported to be able to improve the blood flow by inhibiting platelet aggregation, reducing the inflammatory response, acting on vascular endothelial cells to dilate blood vessels, antithrombolysis, and improving microcirculation, etc. [3, 4]. Thus, it has a potential therapeutic effect on the reduction of ISR occurrence after PCI. However, there is still some controversy regarding the therapeutic effect of alprostadil in improving ISR after PCI, and most of the clinical studies of alprostadil focused on its effect on reducing contrast-induced nephropathy after PCI [5, 6]. Therefore, this study collected and summarized the literature and conducted a meta-analysis, aiming to clarify the effect of nanoliposome alprostadil in the perioperative period of PCI on the ISR within six months to one year after the operation.

## 2. Methods

### 2.1. Strategy for Data Collection

We searched for studies that used nanoliposome alprostadil treatment after PCI in the patients with CHD. Reports were searched in PubMed, Embase, the Cochrane Library, and other databases and network search engines such as Google Scholar in English, as well as the Wanfang database, CNKI database, Weipu database, and other databases in Chinese. Keywords used for the search were as follows: ((Alprostadil) or (Prostaglandin E1)) AND ((coronary) AND ((stent) or (restenosis))), and the research strings with syntax in each search engine were listed in detail (Supplementary information Appendix 1). The period for publication searched ended on October 30, 2022. The nanoliposome dosage form of alprostadil was screened by its description and dosage form of 10 *μ*g for intravenous injection or drip. The main databases were searched initially and then screened by reading abstracts and achieving full-text articles. Records were searched with no language and date restrictions.

### 2.2. Inclusion and Exclusion Criteria

We included publications that (i) were prospective controlled studies among patients with CHD diagnosis (i.e., including ACS and CCS), (ii) reported patients who were subjected to nanoliposome alprostadil treatment after PCI with drug-eluting stent placement, (iii) reported the occurrence of ISR after PCI as the primary endpoint of treatment, (iv) provided basic demographical statistics and the follow-up duration of 6 to 12 months, and (v) were full-text articles. We excluded studies that (i) included patients who could not receive the adjunctive medical therapies such as DAPT or MAPT, (ii) lacked the necessary endpoint as above, or (iii) were designed as preclinical studies, protocols, reviews, or case reports.

### 2.3. Data Extraction

We obtained the full text of 5 articles at the end after screening. Data were extracted from the included studies independently, settling any discrepancies by discussion. 3 researchers completed this work. Two of them initially performed the bibliographic research and data extraction separately, and then, the third researcher checked the extracted results and initiated a discussion when necessary. The collected information contained first author, year of publication, type of trials, number of objects, demographic characteristics of objects, treatments in different groups, primary and secondary endpoints, and the length of follow-up.

### 2.4. Statistical Analysis

Before analyzing, we collected and unified the data from different reports in each study to assess the efficacy of alprostadil treatment after PCI in the patients with CHD. Review Manager software (version 5.3) was used to describe the risk of bias in each study, unify the data from each article, and perform meta-analysis finally. The positive ratio of each endpoint was calculated as rates and then pooled together. We performed the meta-analysis for the efficacy of alprostadil treatment after PCI of patients with CHD with 95% confidence interval (95% CI) and estimated the statistical heterogeneity among studies using the *Q* test and *I*^2^ statistics. *P* < 0.1 in the *Q* test or *I*^2^ > 50% was considered as substantial heterogeneity, and then the random-effect model was used. Otherwise, the fixed-effect model was performed. The funnel plots were used to describe the potential publication bias. The sensitivity analysis was then performed to determine the robustness of the overall treatment effects as the sample size in the included studies was relatively small.

## 3. Results

### 3.1. Selection of Studies

**Figure 1:**
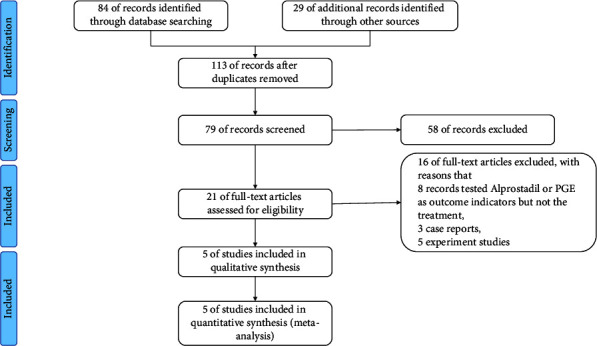
Flow diagram for selection of studies treating patients with conventional treatment or together with alprostadil treatment (figure created by Review Manager software version 5.3, The Nordic Cochrane Centre, and The Cochrane Collaboration, 2014).

We initially identified 113 articles, 84 of which were searched from the main databases (i.e., PubMed, Embase, Cochrane Library, Wanfang database, CNKI database, and Weipu database), and the remainder were found in other sources such as Google Scholar. After duplicates were removed, 79 articles remained for further screening. Totally, 58 records were excluded, and 21 articles were screened to achieve full text for eligibility assessment. Then, 16 of the full-text articles were excluded, with the reasons that 8 records tested alprostadil or PGE as outcome indicators but not the treatment, 3 records were case reports, and 5 records were preclinical studies. Then, 5 studies were included in qualitative synthesis, and these 5 studies [3, 7–10] with full text achieved were included for the final meta-analysis, and the details of the 5 studies are shown in Table 1. Besides, Figure 1 shows the search process.

### 3.2. Characteristics and Baseline of the Included Studies

Totally, this review included 5 prospective controlled studies (4 studies of randomized controlled trials (RCT) and 1 nonrandomized contemporaneous controlled trial) with 463 subjects pooled together, and the characteristics and baseline of which are summarized in Table 1. 3 studies enrolled patients with ACS only [8–10], while the other 2 studies enrolled both ACS and CCS patients [3, 7]. Patients in the control group were 228 in the pooled studies, compared with 235 patients in the test group, and all the patients received PCI with drug-eluting stent placement. Patients in the control group were allocated with conventional treatments (i.e., antiplatelet, ventricular rate control, blood lipid lowering, vasodilator, blood pressure lowering, and blood sugar lowering), while the test group were treated with conventional treatment added with 10 *μ*g alprostadil intravenously. The observation period lasted from 6 to 12 months, and the primary endpoint ISR was diagnosed by symptoms and coronary angiography. The number of males and females involved in this analysis and the number of patients with hypertension, diabetes mellitus, hypercholesterol history in each report are shown in Table 1.

### 3.3. Quality and Bias Assessment

**Figure 2:**
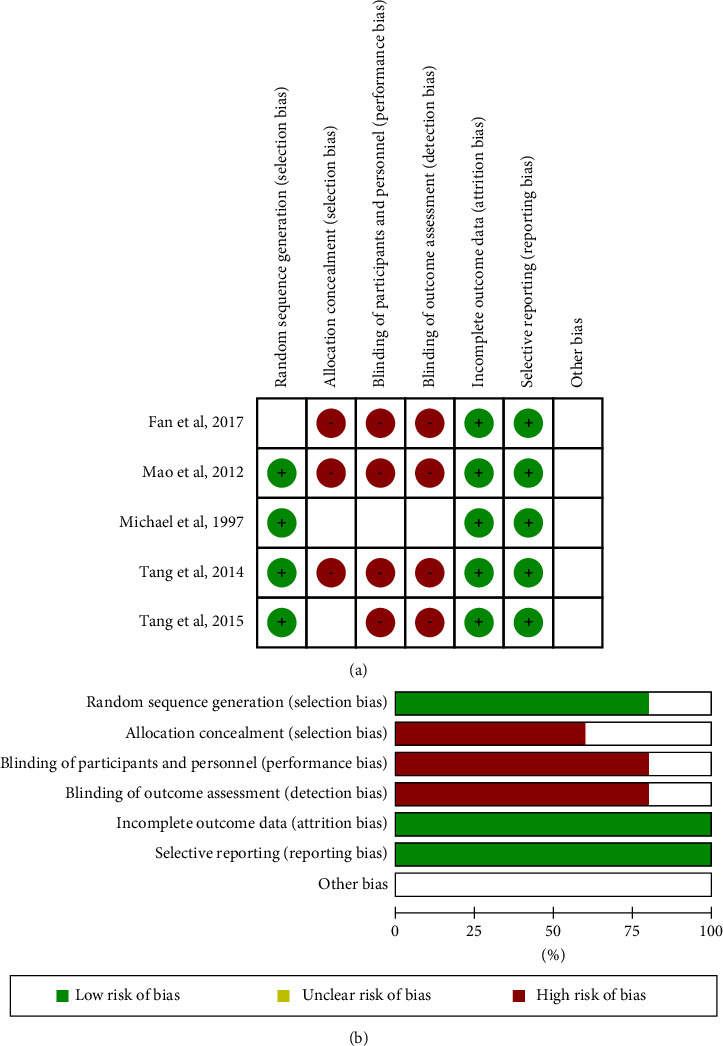
Quality and risk of bias assessment of the included studies. Risk of bias in each study (a); the summarized result (b) (figure created by Review Manager Software version 5.3, The Nordic Cochrane Centre, and The Cochrane Collaboration, 2014).

This review included 6 reports of RCT studies. We assessed the quality and bias of each study using the evaluation form in Review Manager 5.3 software. The risk of bias in each study is shown in Figure 2(a). The summarized results showed that allocation concealment (selection bias), blinding of participants and personnel (performance bias), and blinding of outcome assessment (detection bias) were reported as high risk of bias, while the incomplete outcome data (attrition bias) and selective reporting (reporting bias) were reported as low risk of bias (Figure 2(b)).

### 3.4. Efficacy Analysis of Alprostadil to ISR after PCI in Clinical Outcomes

**Figure 3:**
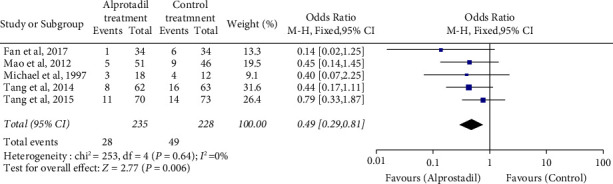
Forest plots of occurrence of in-stent restenosis in patients treated with conventional treatment or added with alprostadil treatment (figure created by Review Manager Software version 5.3, The Nordic Cochrane Centre, and The Cochrane Collaboration, 2014).

Clinical events, mainly the occurrence of ISR after PCI as the primary endpoint, at the endpoint of 6–12 months were pulled together for meta-analysis. The total number of subjects who completed the follow-up was 463, and the forest plots of clinical outcomes are shown in Figure 3. The primary endpoint, i.e., the occurrence of ISR after PCI, occurred in 11.91% of the alprostadil treatment group (28 from 235 patients) vs. 21.49% of the conventional treatment group (49 from 228 patients), and showed a statistical significance in our pooled data (*χ*^2^ = 7.654, *P*=0.006), while there was no statistically significant difference in all of the separate studies. We observed no statistical methodological heterogeneity among the studies (*P*=0.64, *I*^2^ ≈ 0%). The pooled odds ratio (OR) of the occurrence of ISR was 49% in a fixed-effect model, and the 95% confidence boundary (95% CI) was 29% to 81%. Obviously, the pooled results of OR value favored alprostadil treatment in reducing the occurrence of ISR after PCI.

### 3.5. Publication Bias

**Figure 4:**
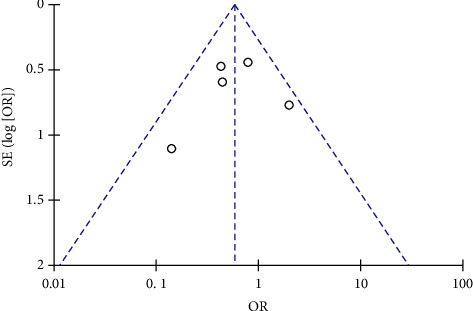
Funnel plots of occurrence of in-stent restenosis from the included studies (figure created by Review Manager Software version 5.3, The Nordic Cochrane Centre, and The Cochrane Collaboration, 2014).

The funnel plot was performed to evaluate the publication bias of the primary endpoint in patients treated with conventional treatment or added with alprostadil treatment (Figure 4). The funnel plot did not show serious publication bias, as most of the studies indicated in the funnel plots were located at the top of the plot and almost evenly distributed.

### 3.6. Sensitivity Analysis

We performed the sensitivity analysis by removing the studies one by one manually, in order to examine the robustness of the overall treatment effect of conventional treatment added with alprostadil treatment. The tested result showed well robustness of the overall treatment effect and showed that the overall effect of alprostadil treatment in reducing the occurrence of ISR after PCI was stable.

## 4. Discussion

This study summarized the efficacy of nanoliposome alprostadil in reducing the occurrence of ISR after PCI in patients with CHD. Meta-analysis in our study favored the clinical add-on effect of alprostadil compared with the conventional treatment and showed a statistical significance in our pooled data, while there was no statistically significant difference in all of the separate studies. Thus, our data emphasized the efficacy of alprostadil in the prevention of ISR with statistical significance, and the reason for the separate studies without statistical significance may lie in the small amount of sample size. Besides, the sensitivity analysis showed well robustness of the overall treatment effect with no serious publication bias.

Cardiovascular events after PCI are related to a variety of factors [11], including direct damage to the arterial wall caused by metal-stent release, excessive proliferation and migration of the vascular smooth muscle cells (VSMC), and inflammatory responses caused by the activation of growth factors, which ultimately lead to vascular intimal hyperplasia and ISR [12]. Several reasons caused intimal hyperplasia after PCI [13]. On the one hand, intimal hyperplasia is caused by the operation of stent release and the support force of stent on the blood vessel. On the other hand, the balloon or stent injures the vascular endothelium, leading to the release of growth factors and mitogens in the blood, especially platelet-derived factors, which stimulate VSMC to proliferate and migrate from the media to the intima of the artery wall [14, 15], accompanied by the degradation and accumulation of a large number of extracellular matrix complexes and proinflammatory cytokines [16], which eventually leads to the formation of a proliferative neo-intima and results in artery lumen restenosis [17–19].

Liposome is the most commonly investigated nanostructure used in advanced drug delivery, which was first discovered by Alee Bangham et al.[20]. The biocompatibility and nanosize enable the potential of nanoliposome in a vast range of clinical applications, covering diagnosis and therapy [21]. Nanoliposome enhances the performance of bioactive agents by improving their solubility and bioavailability and the high selection of cell-specific targeting, which could improve the therapeutic efficacy in the target site while minimizing the adverse effects on healthy cells and tissues [22].

Alprostadil, especially its nanoliposome dosage form, has vasodilator and anti-inflammatory effects and can be used for the treatment of CHD in the perioperative period of PCI [23]. Preclinical studies indicate that prostaglandin receptors exist on a variety of inflammatory cells, such as platelets, dendritic cells, fibroblasts, Th2 lymphocytes, and mast cells [24]. Alprostadil inhibits the release of inflammatory factors, and its application after PCI can not only dilate coronary arteries but also effectively inhibit the inflammatory response [25]. In addition, alprostadil also has the functions of antiplatelet aggregation and reduces thrombosis, which can effectively reduce the occurrence of cardiovascular events after PCI [26–28]. Reports also showed that alprostadil increases the level of cyclic adenosine monophosphate (cAMP) in platelets by activating adenylyl cyclase, while inhibiting the expression of thromboxane A (TXA), thereby inhibiting platelet aggregation and preventing thrombosis [29]. Overall, these effects facilitate alprostadil to reduce the occurrence of ISR after PCI in patients with CHD.

## Figures and Tables

**Table 1:** Description of the characteristics of studies involved in analysis.

Study	Study design	Type of PCI	Control group #			Test group #			Treatment		Dose of alprostadil (*μ*g)
			n	Male	Female	n	Male	Female	Control group	Test group	
Zhiwei et al. [7]	Nonrandomized contemporaneous controlled trials	Elective PCI	34	19	15	34	18	16	Conventional treatment	Alprostadil	10
Lirong et al. [8]	RCT	ACS	46	26	20	51	27	24	Conventional treatment	Alprostadil	10
Shechter et al. [3]	RCT	Elective PCI	12	8	4	18	16	2	Conventional treatment	Alprostadil	10
Ming-Xiang and Xiang [9]	RCT	ACS	63	—	—	62	—	—	Conventional treatment	Alprostadil	10
Mingxiang et al. [10]	RCT	ACS	73	43	30	70	43	27	Conventional treatment	Alprostadil	10

(Study)	ISR diagnosis	Hypertension				Diabetes mellitus			Hypercholesterol		
		Control group	Test group			Control group	Test group		Control group	Test group	

Zhiwei et al. [7]	Symptoms and coronary angiography	24	23			10	9		13	12	
Mao et al. 2012 [8]	Symptoms and coronary angiography	36	40			24	26		29	34	
Lirong et al. [3]	Symptoms and coronary angiography	2	8			1	3		2	2	
Ming-Xiang and Xiang [9]	Symptoms and coronary angiography	—	—			—	—		—	—	
Mingxiang et al. [10]	Symptoms and coronary angiography	38	41			31	30		39	35	

## Additional Points

*Limitations and Perspectives*. Based on the meta-analysis, our study indicated that the early application of nanoliposome alprostadil after PCI could effectively reduce the occurrence of ISR. Besides, there was no serious adverse reaction reported during alprostadil treatment, and its safety was relatively good. However, the total number of cases included in this study was relatively small, the follow-up time was relatively short, and only a small number of RCT studies were involved in this meta-analysis. Moreover, the heterogeneity among the included studies also requires more samples to validate the current conclusion when there are more studies reported. Thus, we are glad to include more reports especially RCT studies for improving the quality of this meta-analysis in future.

## Supplementary Materials

Supplementary Materials Appendix 1: research strings with syntax in each search engine.
