# Supplementary material for: Application of Nanoliposome Alprostadil in the Perioperative Period of Percutaneous Coronary Intervention to Reduce In-Stent Restenosis: A Systematic Review and Meta-Analysis
Source: J Interv Cardiol. 2023 May 18;2023:4100197. doi: 10.1155/2023/4100197 (PMC10212678; doi:10.1155/2023/4100197)
Supplement: Supplementary Materials — Appendix 1: research strings with syntax in each search engine. [file 4100197.f1.docx]

**Supplementary information**

**Appendix 1. Research strings with syntax in each search engine**

1. Pubmed

(("alprostadil"[MeSH Terms] OR alprostadil[Text Word]) OR ("prostaglandin E1"[MeSH Terms] OR prostaglandin E1 [Text Word])) AND (("coronary"[MeSH Terms] OR coronary [Text Word]) AND (("stent"[MeSH Terms] OR stent [Text Word]) OR ("restenosis"[MeSH Terms] OR restenosis[Text Word])))

2. Embase

(('alprostadil'/exp OR 'alprostadil') OR ('Prostaglandin E1'/exp OR 'Prostaglandin E1')) AND (('coronary'/exp OR 'coronary') AND (('stent stenosis'/exp OR 'stent stenosis') OR ('restenosis'/exp OR 'restenosis')))

3. Cochrane

((Alprostadil) or (Prostaglandin E1)) AND ((coronary) AND ((stent) or (restenosis)))

4. Wanfang database

((Alprostadil) or (Prostaglandin E1)) AND ((coronary) AND ((stent) or (restenosis)))

5. CNKI database

((Alprostadil) or (Prostaglandin E1)) AND ((coronary) AND ((stent) or (restenosis)))

6. Weipu database

((Alprostadil) or (Prostaglandin E1)) AND ((coronary) AND ((stent) or (restenosis)))

7. Google scholar

((Alprostadil) or (Prostaglandin E1)) AND ((coronary) AND ((stent) or (restenosis)))
